# Oscillatory beta/alpha band modulations: A potential biomarker of functional language and motor recovery in chronic stroke?

**DOI:** 10.3389/fnhum.2022.940845

**Published:** 2022-09-26

**Authors:** Maxim Ulanov, Yury Shtyrov

**Affiliations:** ^1^Centre for Cognition and Decision Making, Institute for Cognitive Neuroscience, HSE University, Moscow, Russia; ^2^Center of Functionally Integrative Neuroscience (CFIN), Department of Clinical Medicine, Aarhus University, Aarhus, Denmark

**Keywords:** stroke, biomarkers, plasticity, excitatory-inhibitory balance, beta/alpha oscillations, top-down control

## Abstract

Stroke remains one of the leading causes of various disabilities, including debilitating motor and language impairments. Though various treatments exist, post-stroke impairments frequently become chronic, dramatically reducing daily life quality, and requiring specific rehabilitation. A critical goal of chronic stroke rehabilitation is to induce, usually through behavioral training, experience-dependent plasticity processes in order to promote functional recovery. However, the efficiency of such interventions is typically modest, and very little is known regarding the neural dynamics underpinning recovery processes and possible biomarkers of their efficiency. Some studies have emphasized specific alterations of excitatory–inhibitory balance within distributed neural networks as an important recovery correlate. Neural processes sensitive to these alterations, such as task-dependent oscillatory activity in beta as well as alpha bands, may be candidate biomarkers of chronic stroke functional recovery. In this review, we discuss the results of studies on motor and language recovery with a focus on oscillatory processes centered around the beta band and their modulations during functional recovery in chronic stroke. The discussion is based on a framework where task-dependent modulations of beta and alpha oscillatory activity, generated by the deep cortical excitatory–inhibitory microcircuits, serve as a neural mechanism of domain-general top-down control processes. We discuss the findings, their limitations, and possible directions for future research.

## Introduction

Around half of stroke survivors face sustained disabilities that impair their quality of life (Schweizer, 2014). Among the most common deficits are motor dysfunctions, which typically become chronic, remaining in more than 50% of adult cases after six and more months post-stroke (Bonita and Beaglehole, 1988). Some of the most drastic stroke consequences are sustained speech deficits known as post-stroke aphasia (PSA). These are observed in around 20–40% of first-episode stroke cases (Berthier, 2005) and have long-term repercussions: even one year after stroke, more than half of these patients still demonstrate various language difficulties. This makes PSA a chronic neurological impairment with serious socio-economic consequences. In this review, we focus on motor and language impairments as the two most common disabling consequences of stroke.

During the first days and weeks after stroke, functional reorganization of cerebral neural networks takes place (Cramer, 2008). This reorganization is characterized by alterations in brain activity patterns, observed both at rest and during task performance (Weiller, 1998; Saur, 2006). These alterations are triggered by the stroke-induced lesion and by its various consequences (Rossini et al., 2003), and reflect neuroplasticity processes (Hallett, 2001; Saur and Hartwigsen, 2012). The latter is an umbrella term that encompasses various structural and functional changes in the brain, including neurogenesis, gliogenesis, axonal sprouting, changes in excitation/inhibition balance, etc. These processes largely subserve the cortical functional reorganization leading to a partial restoration of the damaged neural networks (i.e., reduction of their impairments), and/or compensation of the impaired functions by other areas (Zeiler and Krakauer, 2013). Functional reorganization undergoes three consecutive stages: acute, sub-acute, and chronic. The first two are characterized by spontaneous neuroplastic changes. By the chronic stage, the spontaneous plasticity usually ends, and new patterns of neural activity become established (Rossini and Dal Forno, 2004). In the chronic stage of stroke, further improvements can only be achieved through various interventions, most importantly, behavioral training which promotes experience-induced functional plasticity (Kerr et al., 2011). Though being widely discussed, the links between changes in brain activity patterns and efficiency of functional recovery processes across the different stages remain a matter of debate (Hartwigsen and Saur, 2019).

To date, a variety of rehabilitation techniques have been developed to restore motor (Langhorne et al., 2009; Stinear, 2017) and language (Berthier, 2005; Schweizer, 2014) functions in stroke patients. However, the improvements achieved are quite variable and typically only partial (Zumbansen and Thiel, 2014; Gerstenecker and Lazar, 2019); often, no significant improvements can be attained, and impairments remain chronic. Thus, a major issue in chronic stroke rehabilitation is the efficiency of intervention-based recovery and the factors that can determine/influence it. Considering the prevalence of stroke consequences, addressing this issue is critical for the well-being of millions of patients worldwide.

This, in turn, entails the need for reliable recovery biomarkers, i.e., measurable parameters of brain activity that could predict clinical and behavioral outcomes (Bernhardt et al., 2016) and reflect the efficiency of rehabilitation at the brain level (Stinear, 2017). However, this issue remains mostly unresolved for chronic stroke, especially in the field of chronic PSA research. Most relevant works provide only structural biomarkers of recovery (Boyd et al., 2017). For instance, one model that may, to a degree, predict the recovery outcomes in PSA is PLORAS – Predicting Language Outcome and Recovery after Stroke (Price et al., 2010; Hope et al., 2013). However, biomarkers of functional recovery in chronic stroke that could rely on or reflect the brain activity patterns associated with training-induced improvements are still lacking.

Neurocognitive impairment and recovery processes in stroke occur at the interlinked physiological (i.e., neurons and networks) and functional (i.e., cognitive functions and overt behavior) levels. Particularly, the changes in the neural excitability processes within the surviving neural networks (Carmichael, 2012) might reflect these networks’ functional efficiency (i.e., amount of behavioral and cognitive deficit). The cortical excitability (or cortical excitation–inhibition balance) is a complex neural phenomenon that has been shown to play a crucial role in neural information processing across healthy and clinical populations (Yizhar et al., 2011; Sohal and Rubenstein, 2019; Iascone et al., 2020), with various factors contributing to its generation and dynamics. Among them the main factor is GABA (gamma-aminobutyric acid) and glutamate signaling balance in the brain’s neural networks (Buzsáki et al., 2007). In stroke, the GABAergic and glutamatergic systems are affected by a set of neurophysiological events that occur in perilesional tissues in the first days and weeks after the stroke onset (Lipton, 1999). These events particularly include a reduced GABA-uptake and a release of glutamate from the damaged cells. Consequently, an extremely increased NMDAR-driven (*N*-methyl-D-aspartate receptors) glutamatergic excitatory activity of pyramidal neurons in the perilesional areas becomes threatening to the preserved tissues—so-called “excitotoxicity” phenomenon (Lai et al., 2014). Additionally, the increased extracellular GABA stimulates the extrasynaptic receptors, providing prolonged tonic inhibition. As a result, the areas adjacent to the lesion become excessively inhibited, and this inhibition, whilst initially protective, might hinder functional recovery at later stages (Clarkson et al., 2010; Clarkson, 2012). Furthermore, some studies indicate that the opposite process, a release of this increased inhibition, is associated with motor stroke recovery that is a result of the functional neural networks, plastic reorganization (Ward, 2017).

In a healthy brain, one of the possible mechanisms to control the cortical excitation level across local and global neural networks is based on the synaptic interactions within microcircuits comprised of excitatory glutamatergic pyramidal cells and inhibitory GABAergic interneurons (Esmaeili et al., 2022). More precisely, the firing rate produced by the deep layer pyramidal cells (i.e., cortical output), depends on the balance between excitatory and inhibitory synaptic inputs that these cells receive (Taub et al., 2013). Pyramidal neurons’ axons form association fibers connecting distant cortical areas and thus contributing to the excitation–inhibition balance across wide brain regions (Deco et al., 2014). This microcircuit interaction is an important neurophysiological mechanism that controls the excitation–inhibition balance across cortex (le Roux et al., 2006). Recent study (Zhou and Yu, 2018) has shown that the balance established as a result of excitatory–inhibitory synaptic interactions determines the efficiency of the sensory information processing within the cortical neural networks. Further, the axons of pyramidal neurons form association fibers connecting distant cortical areas; thus, the microcircuit interaction contributes to the excitation–inhibition balance across wide brain regions (le Roux et al., 2006; Deco et al., 2014). In turn, an imbalance of cortical excitation and inhibition is common for various neural and psychiatric disorders, although the underpinnings of this imbalance may vary (Sohal and Rubenstein, 2019). Importantly, in the present context, pharmacological interventions targeted at modulating cortical excitation and inhibition in stroke patients were shown to drive behavioral recovery after stroke (Carmichael, 2012). Hence, the neural measures that reflect cortical excitation–inhibition balance and its dynamics associated with better behavioral and cognitive performance may potentially have a high diagnostic and prognostic validity as functional recovery biomarkers in stroke.

It therefore appears important to identify such neural measures that could detect excitatory–inhibitory imbalance in functional neural networks using standardized methods. Non-invasive neurophysiological techniques, such as MEG (magnetoencephalography) and EEG (electroencephalography), sensitive to postsynaptic processes in cortical microcircuits formed by reciprocally connected glutamatergic pyramidal and GABAergic inhibitory interneurons, theoretically may provide means to track such dynamics indirectly (Muthukumaraswamy, 2013). The prime candidates for this are the beta (∼13–30 Hz) as well as alpha (∼8–13 Hz) neural oscillations. Although, oscillatory events in other frequency bands, such as gamma and theta, might also depend on the GABAergic activity (Hall et al., 2010; Buzsáki and Wang, 2012; Pignatelli et al., 2012), beta and alpha oscillatory processes are widely observed in different conditions in various cognitive and behavioral tasks (Wang, 2010). Particularly, beta oscillatory events are tightly linked to the motor processes (Kilavik et al., 2013; Rossiter et al., 2014). Beta and alpha oscillations are associated with different aspects of speech functioning (Piai and Zheng, 2019). However, in post-stroke functional recovery not only task-dependent, but also resting-state oscillatory dynamics, especially in beta and alpha bands, might play a crucial role (Dubovik et al., 2012).

Whereas the mechanisms underpinning beta and alpha neural oscillations are subject of a debate (Spitzer and Haegens, 2017), one widely accepted view of cortical beta activity suggest that it is generated by spiking interactions within local microcircuits composed of the interconnected excitatory and inhibitory neurons (Jensen et al., 2005). The inhibitory synaptic GABAergic projections of the interneurons on the pyramidal neurons modulate the excitatory spiking rate the latter cells produce. This architecture makes beta oscillations particularly optimal for composing both local neural assemblies and long-rage cortical communication. An alternative model suggests that beta oscillations might stem from cortico-basal interactions, as has been demonstrated in studies of Parkinson’s disease patients (McCarthy et al., 2011). These two views are not mutually exclusive and have to some extent been combined within a model which suggests that transient neocortical beta oscillations might be produced via synchronization of excitatory synaptic bursts and modulated by subcortical or thalamic inputs (Sherman et al., 2016). In other words, different models of beta activity suggest mechanisms aimed at the regulation of the local cortical excitability, which, in turn, is functionally dependent on GABAergic-glutamatergic interactions in cellular microcircuits, as reviewed above. Models of alpha activity also suggest that cortical inhibitory mechanisms play a key role in the event-related alpha modulations in various cognitive tasks (Klimesch et al., 2007; Klimesch, 2012). On the one hand, the feedback GABAergic synaptic connections of inhibitory interneurons formed on the excitatory pyramidal cells should reduce the effects of the excitatory inputs; on the other hand, GABA-signaling might play an important role in generating alpha activity via a pulsed phasic inhibition provided by interneurons through their synaptic connections with pyramidal neurons (Jensen and Mazaheri, 2010).

Hence, though models of oscillatory activity generation vary, many of them emphasize the role of the GABA-driven inhibitory processes within cortical microcircuits, both for beta and alpha activity. This inhibition controls the firing rate produced by excitatory pyramidal neurons and transferred via their connections, thereby affecting excitability level both locally and in distributed networks. The local generators of beta and alpha rhythms are mostly placed in the deep cortical layers, primarily layer 5 (Lee et al., 2013; Fries, 2015). From there they give backward synaptic projections to the superficial layers (layers 2/3). In some models, these interlaminar projections are assigned an important role in the local cortical communication (Bastos et al., 2018). Particularly, these projections provide suppressive impact on the superficial layers via beta- and alpha-synchronization. This impact is considered as a possible mechanism for top-down control of domain-general neurocognitive processes, such as working memory manipulations (Miller et al., 2018), crucial for complex behavioral and cognitive tasks, e.g., learning new skills. In line with that, the local generator models suggest that dynamic beta synchronization across cortex might reflect formation of distributed neural ensembles and interareal communication processes within them (Spitzer and Haegens, 2017). The oscillatory activity within such cellular ensembles, flexible, robust, and sustainable, might be also considered as neural mechanism supporting working memory processes. This generally goes well in line with the existing evidence that changes in beta as well as alpha oscillatory processes are domain general and might be associated with behavioral changes in performance across motor, cognitive and speech tasks. These suggested links between brain functions and neural excitatory–inhibitory dynamics require more detailed inspection as they reflect the neural mechanisms that might be (1) common for motor and language functioning, and (2) potentially significant for the post-stroke recovery.

Motor-related oscillatory activity in beta band, registered by EEG or MEG during voluntary movements, shows certain event-related dynamics (Pfurtscheller and Lopes da Silva, 1999). This dynamic includes pre-movement decrease of beta power over the motor cortical areas, so-called “event-related desynchronization” (ERD), and the post-movement increase of beta power, “event-related synchronization” (ERS), also called “post-movement beta rebound” (PMBR). Interestingly, these movement-related beta oscillatory patterns show a high intraindividual reliability (Espenhahn et al., 2017). Beta activity found in sensorimotor areas plays multiple roles in voluntary movements, including sensorimotor integration, anticipation, and control processes [see Schmidt et al. (2019) for review] At the same time, beta activity observed outside sensorimotor cortex during voluntary movement tasks, particularly in prefrontal areas, might be associated with the executive control of movements that relies on the working memory and attentional systems.

A review by Piai and Zheng investigated the roles that different types of oscillatory activity play in language tasks performance (Piai and Zheng, 2019). It emphasized that beta/alpha processes observed over inferior parietal, temporal and frontal areas during speech tasks might be associated with context-driven speech production processes. The latter might be due to the role these regions, especially lateral prefrontal cortex, supplementary and pre-supplementary motor areas, anterior cingulate cortex, play in the executive control processes. This points to the role of beta activity as a mechanism subserving top-down control for the language function, including lexical-semantic processes (i.e., categorization and retrieval), syntactic processes (i.e., parsing and binding), and domain-general processes, attention, working memory and executive control, involved in speech performance (Weiss and Mueller, 2012).

Such findings demonstrate that beta and alpha oscillatory events might be tightly linked with both voluntary motor and language processes, as well as with the control of complex behaviors more generally. Indeed, the control of motor actions and the domain-general processes (above all, top-down working memory control) have much in common: both rely on the prediction-driven and context-dependent modulations of the current behavioral output (Engel et al., 2001). At the brain level this top-down control is supported particularly by the frontoparietal cortical networks, crucial for working memory maintenance, attention allocation, motor planning and experience-dependent learning (Ikkai and Curtis, 2011; Zehetleitner et al., 2012).

This link between motor and language functions goes well in line with embodied cognition theories postulating that two systems are intrinsically intertwined (Pulvermüller, 2005; Jenson and Saltuklaroglu, 2021). Within this approach, cortical networks with nodes in sensory and in motor areas subserve a wide range of the complex cognitive functions, including attention, working memory, language and voluntary behavior (Pulvermüller et al., 2014). The concept of the tight neural links between motor and language functions is also supported by clinical data on the post-stroke motor and language recovery (Gialanella and Ferlucci, 2010; Ginex et al., 2017). For instance, motor performance training in patients with post-stroke hemiparesis turned out to improve their speech scores as well (Harnish et al., 2014), whilst another study demonstrated that a long-term movement therapy through goal-directed tasks leads to both motor and language improvements (Arya and Pandian, 2014). Furthermore, electric stimulation of the primary motor cortex (M1) in 18 patients with PSA improved word-retrieval abilities (Meinzer et al., 2016), whereas a recent cohort study provided evidence that motor and language recovery in stroke patients with both movement impairments and aphasia interact after intensive motor and language rehabilitation (Ginex et al., 2020). Systematic investigation of such interactions points to the crucial role of domain-general abilities as predictors of motor recovery in patients with PSA. At the neural level, this putative connection between language and motor functions (such as manual movement) could be their shared mechanism of sequential action supported by pars opercularis (Brodmann area 44, part of Broca’s area), which may, in turn, stem from the evolutionary development of spoken language from gestural motor activity (Fadiga et al., 1995; Anderlini et al., 2019).

Still, the exact neural mechanisms driving both motor and language recovery in stroke remain mostly obscure. Particularly, for chronic stroke recovery an important issue is the potential impact of behavioral training interventions on the neural plasticity mechanisms (Hallett, 2001; Kerr et al., 2011). The studies in healthy controls (Bavelier et al., 2010) show that excitatory–inhibitory balance within functional neural networks might be a factor of cognitive and motor training efficiency in healthy individuals. Interestingly, these training-induced modulations of excitatory–inhibitory balance are found both for lower-order sensorimotor networks and higher-order ones, associated with domain-general cognitive functions. Computational models (Ingrosso and Abbott, 2019) of spiking recurrent neural networks indicate that excitation–inhibition balance in such networks might be trained and, as a result, might produce more optimal input–output association during task solving. In clinical studies, normalization of functional neural networks’ activity via induction of synaptic plasticity is a recovery mechanism used in treatment of neurological and mental disorders, for instance in neurorehabilitation techniques based on inhibitory control training (Spierer et al., 2013). More generally, the behavioral experience relevant to the specific impairments and delivered to patients using optimal rehabilitation training techniques induces experience-dependent synaptic plasticity processes (Warraich and Kleim, 2010). The findings on application of this principle in post-stroke recovery (Nie and Yang, 2017) show that behavioral training leads to an enhancement of the synaptic transmission through an increased receptor proteins expression.

Considering the experience-induced plastic reorganization processes in the neural networks supporting domain-general top-down control processes (involved in both motor and language functions), one may suggest that they will involve alterations of the excitatory–inhibitory balance within the neural networks supporting these abilities. Such training-induced alterations might change synaptic efficiency in the microcircuits composed of cortical pyramidal neurons, interneurons, and their connections. Thus, measuring beta and alpha oscillations, produced by these microcircuits, might be a way to probe the experience-induced dynamics of excitation–inhibition balance in motor and language stroke patients as they undergo recovery and rehabilitation. Though several studies have tackled this idea [see Ward (2017) for review], the impact of behavioral training on the functional plasticity processes and the excitatory–inhibitory balance within sensorimotor and especially higher-order cognitive cortical areas have not been studied systematically in chronic stroke patients, and particularly little is known regarding PSA recovery.

To sum up, stroke patients’ studies suggest that functional recovery after stroke depends on the cortical excitation–inhibition balance restoration. This balance might be significantly influenced by the interaction of excitatory pyramidal cells and inhibitory interneurons. The synaptic connections strength in these microcircuits impacts both local and distributed cortical information processing efficiency. Existing models suggest that interactions within such microcircuits might be the source of beta and alpha oscillatory activity. Moreover, beta and alpha activity frequency bands are also considered as a channel of the intracortical communication, providing domain-general top-down control of the working memory processes. Studies of voluntary motor activity and speech processes, in turn, demonstrate that particularly beta activity plays a common role in both of these functional domains, which possibly reflects the involvement of top-down control processes in these complex behavioral and cognitive abilities (see Chart 1 in Supplementary material). Crucially, clinical data in stroke patients show that both motor and language recovery frequently occur together, which also indicates a link between the two processes, possibly through the common domain-general mechanisms. As the latter might rely on the intracortical excitatory–inhibitory interactions, behavioral interventions that induce synaptic plasticity in the cortex could be the tool that restores functional excitation–inhibition balance in a stroke-affected brain. Based on this, two main suggestions could be made. The first suggestion is that beta and alpha oscillatory activity modulations might be candidate biomarkers of the functional recovery in chronic motor and language stroke, as they reflect the dynamics of the cortical excitation–inhibition balance, associated with domain-general abilities. The second suggestion is that training-induced modulations of the beta/alpha oscillatory activity within distributed functional neural networks might predict motor and language rehabilitation outcomes in chronic stroke patients.

Below, in a brief overview of data from several available studies, we examine these suggestions and ask whether modulations of oscillatory activity, particularly in beta and alpha bands, might provide the much-needed biomarkers of functional recovery and behavioral training efficiency in chronic stroke. We start with somewhat better-studied motor stroke recovery before continuing to much less understood PSA. For both systems, we will review selected experimental evidence available for resting state and active task conditions to see if oscillatory processes might be candidate biomarkers of efficient speech and motor recovery in chronic stroke patients and possibly serve as a correlate of experience-induced improvements. Finally, open issues and possible further directions will be discussed.

## Resting-state oscillations and chronic motor stroke recovery

First, we review several studies on the resting state oscillatory activity as a recovery correlate in chronic motor stroke patients. A recent study by Hordacre and colleagues employed resting-state EEG functional connectivity analysis to identify oscillatory patterns associated with better motor functioning in 36 chronic stroke patients compared with 25 healthy controls (Hordacre et al., 2020). Transcranial magnetic stimulation (TMS) was used to obtain motor-evoked potentials (MEPs) as a measure of corticospinal tract integrity. The interhemispheric resting-state functional connectivity (RFSC) was evaluated for the beta oscillatory band (14–30 Hz) between left and right scalp electrodes, positioned approximately over sensorimotor cortices of both hemispheres. The connectivity measures turned out to be different in two subgroups of patients: those who successfully demonstrated MEP in response to the TMS stimulation at a particular resting motor threshold (MEP+) had a stronger beta functional connectivity than those who did not (MEP−). Moreover, this beta-band resting-state functional connectivity correlated positively with cumulative behavioral measurements of upper limbs functions. The authors emphasized that this correlation was observed primarily in MEP+, but not in MEP− patients. When analyzed together, the measures of beta oscillatory functional connectivity over sensorimotor cortex and corticospinal tract integrity improved the regression model explaining the upper limb functioning efficiency. In other words, the studied beta oscillatory processes are related to sensorimotor cortical activity and its functional connectivity and might be considered as correlates of motor functional recovery.

Thibaut and colleagues investigated the link between motor abilties in chronic stroke and resting-state oscillations in EEG (Thibaut et al., 2017). Oscillatory power in high alpha (10–13 Hz), low beta (13–20 Hz), and high beta (21–30 Hz) bands were correlated with motor functioning scores in a group of 55 chronic stroke patients. Stepwise regression analysis established that, among these bands, only high-beta power increase predicted motor performance. Importantly, the correlations had opposite directions in the two hemispheres. In the affected hemisphere, increased high beta (21–30 Hz) power over the central electrodes correlated negatively with Fugl-Meyer motor scale measures (Gladstone et al., 2002) and with motor threshold scores (measured using TMS), while in the unaffected hemisphere high-beta power increase correlated positively with better motor performance. The authors suggested that the excess of beta power over central electrodes might reflect a lesion-induced excitability imbalance in the bilateral sensorimotor areas. Functionally, this imbalance might reflect pathological reorganization of the sensorimotor cortex and/or increased difficulty of the task for stroke patients. Notably, such a pattern is typically observed in motor studies of older populations (Gola et al., 2012) and might reflect a behavioral compensatory strategy related to decreased efficiency of neural networks in aged individuals and their maladaptive functioning in stroke patients. These results support the idea that cortical excitability level within functional neural networks might be measured non-invasively using an EEG resting state beta power estimate. This measure shows differential roles of affected and unaffected nodes within this network and might therefore be used as a biomarker of recovery efficiency at the chronic stage of stroke. The authors emphasized that these resting state oscillatory measures might reflect general (in)efficiency in cortical processing that could impact motor task performance as well. The latter, however, must be verified in active task studies.

Another interhemispheric asymmetry measure was employed by Saes and colleagues, who obtained resting-state EEG data from 21 chronic stroke patients with upper-limb paresis and from a matched control sample (Saes et al., 2019). They found that the spectral power in low-frequency bands: delta (1–4 Hz) and theta (4–8 Hz) was greater in the affected comparing to unaffected hemisphere. The pairwise BSI measure (brain symmetry index, a measure that reflects the spectral power asymmetry in pairs of homologous EEG channels) was stronger in stroke patients compared to controls in delta and theta bands. Moreover, BSI for these bands negatively correlated with motor performance measured using Fugl-Meyer scores. The results may reflect stroke-caused cortical disorganization of the sensorimotor system leading to deficits in selective motor control. Indeed, low-frequency oscillations are known to be a typical marker of various cortical dysfunctions, considered to reflect an increased protective inhibition of the lesioned areas (Butz et al., 2004).

The available data on the role of the resting-state oscillations in chronic motor stroke recovery are quite limited and heterogeneous (see the Supplementary Table 1). Different measures are used to estimate the contribution of the rhythmic activity into motor performance. Moreover, authors put different frequency bands in the focus of their analysis. However, some tentative conclusions might be drawn out even on such a limited basis. First, these studies show that motor-related measures associated with cortical excitation–inhibition balance, particularly beta-band activity, might be detected in the resting state. Moreover, the higher-frequency beta oscillations may be associated with functional mechanisms driving motor performance in chronic stroke patients. This is supported by data on functional connectivity as well as by beta power correlations with behavioral improvements (Thibaut et al., 2017; Hordacre et al., 2020). Notably, the association between higher-frequency oscillatory dynamics and behavioral recovery might vary across the hemispheres, that may point to the different roles that the affected and the intact hemispheres play in recovery (Thibaut et al., 2017). In contrast, the data on low-frequency activity suggest that excessive theta and delta might be a correlate of dysfunctional performance. This goes in line with the general notion of the low-frequency oscillatory activity playing a deleterious role in recovery (Butz et al., 2004), whereas a decrease of low-frequency inhibition in perilesional areas might cause an increase of excitability in them, shifting it to the premorbid level. Hence, these resting-state patterns and their correlations with behavior might also reflect different reorganization efficiency of the neural networks supporting motor task performance. Still, this efficiency might be established only based on the active paradigms and training-induced dynamics which were not addressed in these studies.

## Task-dependent oscillatory modulations and chronic motor stroke recovery

Relations between motor task performance of chronic stroke patients and task-dependent oscillatory modulations were studied by Shiner and colleagues, who used MEG to record movement-related beta activity (13–30 Hz) in chronic motor stroke patients (Shiner et al., 2015). The results showed that elongated pre-movement beta-power decrease (ERD), most prominently over bilateral primary motor and premotor cortices, was linked with poorer motor performance. In turn, elongated post-movement beta-power increase (ERS) was associated with better performance. Furthermore, stronger amplitude modulation (both for ERD and ERS) and its greater lateralization to the affected hemisphere were associated with better motor performance. The authors concluded that not only the strength of ERD/ERS but also the ability for dynamic modulation of beta power might play an important role in recovery, as it might reflect the extent of residual ability for motor control. However, the impact of functional motor training on modulating these neural processes was outside the scope of this study.

Such training effects were investigated by Espenhahn and colleagues, who showed an association between motor skill acquisition through goal-directed training and changes in beta oscillatory power in the sensorimotor cortex in chronic stroke patients (Espenhahn et al., 2020). At first, patients’ beta activity was less responsive to training, in comparison to healthy controls. However, post-movement beta and alpha (10–25 Hz) rebound amplitude registered over the affected sensorimotor cortex immediately after training (beta/alpha-ERS, recorded using EEG at central and centroparietal electrode sites) turned out to be a predictor of post-training performance as it showed a positive correlation with motor task measurements. Importantly, it predicted not the immediate performance, but delayed improvements obtained 24 h after the training, suggesting that beta/alpha activity may reflect training-induced plasticity.

In turn, Wilson and colleagues found other oscillatory power correlates of training-induced plasticity in motor stroke rehabilitation in a case-series MEG study (Wilson et al., 2011), which combined intensive goal-directed motor training with peripheral nerve stimulation. The authors investigated changes in patterns of movement-related beta (16–28 Hz) and gamma (74–86 Hz) activity in the primary motor cortex and SMA. They found a reduction of movement-related beta-ERS amplitude in the precentral gyrus bilaterally and of gamma-ERS in the affected hemisphere after rehabilitation. Moreover, post-therapy motor improvements (measured using various clinical scales) significantly correlated with both lower beta- and gamma-ERS in the precentral gyrus of the affected hemisphere. A possible explanation of the opposite beta-modulation patterns found in these two studies, apart from peripheral stimulation, might be the duration of the training, which was much greater in this study than in the previous one (Espenhahn et al., 2020). Hence, the changes observed by Wilson and colleagues might be a marker of longer-scale plasticity associated with later stages of motor skills acquisition. Interestingly, the authors also mentioned post-rehabilitation effects in the affected SMA where the beta-ERS reduction was observed, although no correlations with clinical improvements were reported.

In addition to focal effects in the lesioned systems, studies of experience-induced plasticity in motor stroke rehabilitation also address network processes beyond the local lesion or perilesional areas. In an MEG study by Buch and colleagues, movement-related beta and alpha oscillations were analyzed as parts of sensorimotor mu-rhythm (Buch et al., 2012). This study used a specific training approach to improve the motor skills of severe chronic stroke patients. Their grasping abilities were trained using a brain-computer interface that provided biofeedback on the motor mu-rhythm modulations in alpha (9–12 Hz) and beta (20–24 Hz) bands. The goal of the training was to voluntarily modulate mu-rhythm power; successful trials were followed by orthosis device posture shift. Regression analysis showed that training-induced ability of volitional modulation of mu-rhythm power during motor task performance was positively associated with better recovery. Further, structural integrity measures revealed that training success relied on both structural and functional integrity of the distributed networks. These networks included not only the primary motor areas, but also the bilateral frontoparietal cortical areas, structurally connected via superior longitudinal fasciculus. Functionally these networks support controlled visuomotor integration during motor planning and imagery. Remarkably, the link found was individually variable.

Individual variability of recovery outcomes associated with oscillatory dynamics was inspected using EEG in chronic motor stroke patients who underwent a rehabilitation program based on a robotic orthosis device training guided by self-modulations of sensorimotor rhythm (Ray et al., 2020). The results showed that alpha (8–12 Hz) ERD over bilateral central and parietal electrodes during movement attempts correlated significantly with motor improvements. Interestingly, before rehabilitation, the patients’ group was found to be heterogeneous and included two subgroups: those with a relatively strong vs. relatively weak ipsilesional alpha-ERD. The former group showed improvements correlated with the increase of ERD over centroparietal electrodes after training, while the latter group demonstrated the opposite trend of centroparietal ERD decrease with improvements. Moreover, there was a general trend of better clinical improvements correlation with alpha-ERD shift toward ipsilesional hemisphere This finding was interpreted in the following way: different patients’ subgroups employed different motor control strategies in the orthosis task depending on the initial excitatory/inhibitory imbalance between the hemispheres. Moreover, this initial interhemispheric alpha activity imbalance had a prognostic value: a larger motor improvement occurred in patients when ERD was progressively larger over the affected hemisphere in comparison with the unaffected one. This result is well in line with the correlations between motor functioning and resting-state oscillations (Thibaut et al., 2017) mentioned in the previous section. In contrast with those findings, these results were reported for alpha, but not for beta oscillatory band; this, however, corroborates previous findings showing that alpha activity synchronization reflects cortical inhibitory processes suppressing irrelevant components of planned motor actions (Pfurtscheller et al., 1996; Klimesch et al., 2007). On the other hand, the authors acknowledged that the lack of beta effects might be an artifact of the analysis strategy which pooled data across the entire trial period whereas beta desynchronization is typically observed at the beginning of the movement only.

The studies of neural oscillations in active motor tasks in chronic motor stroke patients show that movement-related modulations (desynchronization and synchronization) of oscillatory activity centered around beta and alpha bands are associated with the more successful recovery of motor functions in chronic stroke (see the Supplementary Table 1). The oscillatory activity parameters that predict better recovery include power modulations, duration of synchronization and desynchronization and spatial localization of these events. In terms of spatial and power features, generally stronger decrease of oscillatory power centered around beta/alpha band within the affected hemisphere is related to better motor performance (Buch et al., 2012; Shiner et al., 2015; Espenhahn et al., 2020). However, in some cases these dynamics might also affect other frequency bands, i.e., gamma band (Wilson et al., 2011). The specific lateralization pattern may suggest that better recovery occurs in cases when the affected hemisphere is more functionally intact. The greater decrease of beta power in these cases, in line with its inhibitory role suggested by neural microcircuits models (Spitzer and Haegens, 2017), might point to the greater excitability of the affected hemisphere. Hence, this is a possible marker of its larger functional potential. However, comparison of the training interventions studies (Wilson et al., 2011; Espenhahn et al., 2020) shows that, this association is not completely clear and might depend on various factors including training duration, individual patients’ differences, etc. Also, the training-related oscillatory modulations are observed both in affected primary motor areas and in widely distributed neural networks; in addition to beta oscillations, they also involve adjacent frequency bands. Remarkably, the recovery-related dynamics found in these areas might be individually variable (Buch et al., 2012; Ray et al., 2020). Still, the general pattern of extralesional areas involvement into the recovery-related oscillatory activity in beta and alpha bands is consistent across most of the studies. This might point to the compensatory role these areas and processes play in the recovery, probably associated with domain-general behavioral control abilities and different strategies that individual patients used to overcome their motor deficits.

## Resting-state oscillations and chronic post-stroke aphasia recovery

Following the same logic as above, in this section, we will review a set of studies on resting-state oscillatory activity in relation to chronic stroke recovery, before addressing task-dependent activity in the following section. To link this section with the previous one, we wish to first emphasize the study by Nicolo and colleagues which demonstrated association between oscillatory mechanisms in motor and language recovery (Nicolo et al., 2015). The authors explored resting-state EEG oscillatory dynamics during the recovery of both motor and language functions in stroke patients using coherence connectivity analysis. Patients were tested twice: at the end of the acute/beginning of the subacute stage and at the beginning of the chronic post-stroke stage, with a complex rehabilitation program in-between. At the first recording, there were correlations found in the left hemisphere between behavioral improvements and oscillatory dynamics in the beta band. Motor improvements correlated with the global functional connectivity of the primary motor cortex whereas language improvements correlated with increase in Broca’s area’s global functional connectivity. For the right hemisphere, similar correlations were found in the theta band. In contrast, by the beginning of the chronic stage, the same connectivity measures within the same hemispheres showed negative correlations with corresponding function improvements. As the authors suggested, this change might reflect the return to the close-to-normal pattern of functional oscillatory activity during the chronic stage. Furthermore, theta dynamics in the right hemisphere might reflect interhemispheric reorganization mostly associated with the loss and subsequent restoration of transcallosal inhibition across post-stroke recovery stages. This study also clearly demonstrates that recovery processes, both motor- and language-related, might occur outside the lesioned areas, and that the recovery-related oscillatory dynamics in the lesioned primary areas and compensatory extralesional ones differ at different recovery stages, even within the same bands.

Dalton and colleagues compared resting-state EEG oscillatory power in different frequency bands (delta, theta, alpha and beta) between chronic poststroke aphasia patients and healthy controls (Dalton et al., 2021). Significant differences were found for beta and theta oscillations recorded during the eyes-closed condition: patients demonstrated lower beta (13–30 Hz) and higher theta (4–7 Hz) power than healthy controls. Moreover, the speech comprehension measure used in this study, so-called main concept scores (Nicholas and Brookshire, 1995), demonstrated positive correlations with beta and alpha (8–12 Hz) power in the left hemisphere and negative correlations with the whole-brain theta power. For the eyes-open condition, the same speech measure positively correlated with the beta power (either whole-brain or left-lateralized, depending on the montage), while neuropsychological scores measured using the RBANS test battery (Randolph et al., 2010) showed positive correlations with beta power in the left hemisphere. In this condition patients also demonstrated greater resting state beta power level than controls. Both for eyes-open and eyes-closed conditions, patients’ speech scores showed negative correlations with theta power. The results suggest that the negative correlations of speech scores with theta power might reflect the role of theta activity in maladaptive processes in the lesioned brain, which is strikingly similar to a motor stroke resting-state result reviewed above (Saes et al., 2019). On the other hand, beta power showed a correlation with a complex measure of speech – the main concept scores. Consequently, higher frequency activity was associated with adaptive behavioral dynamics.

Training-induced neural dynamics was also tracked in a single-case study of a chronic PSA patient (Rozelle and Budzynski, 1995). In this study the rehabilitation training procedure employed EEG-based biofeedback targeted at the voluntary decrease of low-frequency theta activity (4–7 Hz) and increase of high-frequency activity in the beta band. Neuropsychological testing after rehabilitation showed improvements in several parameters, including speech fluency, naming abilities, and attention. EEG measures showed the expected decrease of theta activity over the left hemisphere (frontal, central and parietal regions) and an increase of inferior beta-band activity (15–18 Hz) over frontal and midline regions. The authors concluded that their findings indicate that the low-frequency activity decrease, and the high-frequency activity increase are associated with the better recovery.

Meinzer and colleagues specifically focused on training-induced delta-band modulations (Meinzer et al., 2004). A group of chronic patients underwent a 2-week intensive course of Constraint-Induced Aphasia Therapy (CIAT, Pulvermüller et al., 2001; Pulvermüller and Berthier, 2008) with the outcomes evaluated using clinical speech tests (Aachen Aphasia Test, AAT and Token test). Resting-state delta-band activity (1–4 Hz) in the perilesional cortex was used as a measure of neurophysiological changes detected by MEG. Remarkably, different oscillatory patterns were associated with language improvements during the therapy: a subgroup of 16 patients showed an improvement-associated delta-band decrease, whilst in 12 patients’ recovery was associated with the power increase. The authors interpreted this outcome as reflecting different roles of delta-related inhibition in the affected hemisphere, depending on the impairment severity: in mild-to-moderate cases, it might be dysfunctional/counterproductive, whilst in the more severe cases it might be protective and is linked to a compensatory right-hemispheric activity. Notably, unlike the other studies above, this study did not investigate any of the higher-frequency bands.

The limited data on the relations between resting state oscillatory processes and chronic posts-stroke aphasia recovery come from studies with quite heterogeneous designs and different measures of speech abilities (see the Supplementary Table 2). Those of them that focused on training effects also used different rehabilitation procedures (Rozelle and Budzynski, 1995; Meinzer et al., 2004; Nicolo et al., 2015). The results mostly indicate that, similar to the resting-state data in motor studies, the power increase in higher frequency bands, in particular beta, correlates with better recovery, although its distribution varies (Dalton et al., 2021). To reach more precise conclusions, for instance, on the spatial characteristics of these processes, more neuroanatomically precise recordings are still needed. The study by Nicolo and colleagues focused on the resting-state oscillatory dynamics in motor and language recovery together might shed some light on these issues (Nicolo et al., 2015). On the one hand, it demonstrated that the recovery-related oscillatory dynamics is distributed across wide regions and is asymmetrical with respect to the two hemispheres. Moreover, its relation to the functional recovery depends on the recovery stage and probably on the cortical region. Crucially, this study shows both for motor and language recovery that it is associated with the same modulations of oscillatory patterns. The other study that provided more spatially specific data is that by Meinzer and colleagues performed using MEG (Meinzer et al., 2004), which showed that the lower-frequency delta oscillatory activity in the affected left hemisphere manifested different directions of correlations with training-induced improvements across patients. This might depend on the severity of the speech deficits and point to individually specific recovery strategies.

## Task-dependent oscillatory modulations and chronic post-stroke aphasia recovery

Below, we will overview the available limited findings on the role that beta/alpha oscillatory dynamics may play in language recovery in chronic PSA.

Spironelli and colleagues compared high-beta band (21–28 Hz) EEG activity between patients with chronic aphasia and healthy controls in a task with different linguistic demands (Spironelli et al., 2013). Subjects were presented visually with two consecutive words and asked to compare their phonological, orthographic, or semantic features. The analysis showed differences in high-beta activity depending on the subject group, task demands, and a particular set of electrodes. Over the anterior lesioned sites, the high-beta in patients was left-lateralized and had a lower amplitude than in controls for phonological and orthographic tasks. At the central electrodes the same activity was right-lateralized for semantic and phonological tasks and bilateral for orthographic task. In contrast, at the posterior sites, the beta activity was task-independent, its pattern was bilateral in controls and right-lateralized in patients. The posterior high-beta activity also had smaller amplitude in patients than in controls. Moreover, its lower amplitude correlated with better semantic and phonological task performance. Based on this, the authors concluded that the posterior high beta activity might be a correlate of the functional reorganization of bilateral language neural networks in chronic aphasics. Remarkably, this reorganization demonstrates different patterns across anterior/posterior portions of these networks.

The MEG technique was used by Meltzer and colleagues to study a group of chronic PSA patients along with controls (Meltzer et al., 2013). The patients had mild-to-moderate speech deficits (mostly anomic) caused by a single left-hemispheric stroke. The study goal was to map the patterns of beta/alpha activity modulations associated with language recovery in chronic PSA. The authors gave their participants an auditory comprehension task using sentence-picture matching. Sentences varied in their syntactic features, and the contrasts between syntactically different conditions were studied. The results showed an ERD pattern in the 8–30 Hz band, which was more right-lateralized for patients. The increased right-hemispheric activity (i.e., stronger ERD) was found in parietal, temporal, and frontal regions. However, the activity pattern varied across the task performance stages. During sentence comprehension, the beta/alpha-ERD was observed beyond the perisylvian language areas and was mostly bilateral. During a delay period before picture presentation, it became more right-lateralized and involved the right superior, middle frontal gyri, as well as the superior and inferior parietal lobes. Correlational analysis showed that the beta/alpha ERD strength in bilateral posterior temporal and parietal regions correlated with better comprehension during sentence presentation. For the memory delay period, beta/alpha ERD in right superior and middle frontal gyri, superior and inferior parietal lobes correlated with better performance. The strength of ERD over right superior and middle frontal gyri, and superior and inferior parietal lobes correlated with better comprehension scores in chronic aphasia patients. The authors hypothesized that, as the task was challenging for the patients, it required compensatory involvement of additional cognitive resources via activation of the dorsal frontoparietal networks. The latter caused an increased load on domain-general cognitive mechanisms such as short-term working memory, which led to the specific activation pattern observed in the study.

Another MEG study (Kielar et al., 2016) used a different sentence comprehension paradigm: subjects (patients with chronic aphasia and age-matched healthy controls) were asked to judge whether the sentences presented visually were correct or contained semantic or syntactic violations. The patients’ sample was compared with healthy controls. The analysis focused on oscillatory activity modulations across multiple brain regions, including perilesional and contralesional areas in beta and alpha bands (8–30 Hz). The results showed that semantic tasks were associated with smaller beta/alpha-ERD in ventral frontotemporal, anterior temporal and temporo-occipital areas for patients than for age-matched controls. For syntactic tasks, patients showed weaker beta/alpha-ERD than controls in frontal, posterior temporal and dorsal parietal areas. The correlations analysis showed that for semantic tasks, right-hemispheric beta/alpha-ERD (8–30 Hz) in right superior, middle and inferior temporal gyrus correlated with greater performance accuracy. For syntactic tasks, better scores were associated with bilateral task-induced beta/alpha-ERD in temporal areas and in the right temporoparietal cortex, superior temporal gyrus, precuneus, middle frontal gyrus and left inferior parietal lobule. The authors hypothesized that the oscillatory dynamics observed in syntax tasks reflect compensatory involvement of domain-general neurocognitive systems of working memory, executive control and top-down attention, all of which are associated with frontoparietal networks (Palva et al., 2010; Takeuchi et al., 2011; Murray et al., 2017).

A similar result was found by Piai and colleagues, who used EEG and MEG and compared six chronic aphasics with impaired speech comprehension and six healthy controls on a comprehension task performance. The results showed beta/alpha-ERD (8–25 Hz) after the cue sentence presentation and prior to the target picture stimuli. This effect was left-lateralized in controls and right-lateralized in patients; furthermore, it was absent in patients with the lowest performance scores. The authors hypothesized that this ERD effect might be associated with context-driven retrieval aspects of the working memory, supported by the right hemisphere in a compensatory way.

The studies reviewed suggest that task-dependent oscillatory power modulations may show specific power and spatial patterns in chronic posts-stroke aphasia patients and in healthy controls (see the Supplementary Table 2). These modulations affect mostly beta and alpha frequency bands (although, notably, not all studies analyzed the two bands separately). Generally, these studies show that spatially these activity patterns are more right-lateralized in patients in comparison with controls (Meltzer et al., 2013; Spironelli et al., 2013; Kielar et al., 2016; Piai et al., 2017). Functionally, the modulations of these oscillatory patterns in patients might be related to domain-general higher-order cognitive processes. These processes are suggested to play a compensatory role in chronic PSA recovery. Most of the available research suggests that ERD – a decrease of power in beta and alpha bands – is linked to better functional performance. Importantly, this ERD is found mostly outside the lesioned and language-specific perisylvian areas, which, according to the view shared by different studies (Meltzer et al., 2013; Kielar et al., 2016; Piai et al., 2017), may point to the intrinsic involvement of working memory and executive control processes in the recovery. Testing this suggestion seems to be a worthwhile target for future investigations, particularly as a similar pattern also emerges from non-speech motor stroke research overviewed in the previous section. On the other hand, the spatial patterns of beta and alpha modulations associated with better recovery might vary across different tasks: phonological, semantic, syntactic (Meltzer et al., 2013; Spironelli et al., 2013; Kielar et al., 2016). This does not necessary contradict the previous suggestion about the role of the domain-general mechanisms reflected in the beta-alpha modulations in aphasia recovery and might point to spatially different networks supporting the same neural mechanism to compensate for different types of language deficits. Finally, a more detailed inspection of the distinctions between higher and lower frequency bands, especially within the beta band, and their association with training-induced functional recovery, might be beneficial.

## Discussion

As described in the Introduction, the processes of impairment and recovery in stroke are likely associated with the dynamics of cortical excitation–inhibition balance, which is a product of interactions between excitatory and inhibitory cells (Buzsáki et al., 2007). The microcircuits these cell form in the deep cortical layers as well as their proximal and distal projections are, in turn, considered as possible generators of cortical beta and alpha oscillations (Jensen et al., 2005; Klimesch et al., 2007). Moreover, the interlaminar inhibitory projections from these circuits in the deep cortical layers to the superficial may underpin a possible mechanism of top-down control (Miller et al., 2018). The top-down control is an ability common for various cognitive and behavioral functions, including voluntary movements and speech. Considering the clinical data showing that both motor and language functions are frequently impaired (and recovered) together in stroke, we suggested that top-down control might be the shared mechanism driving the recovery of both functions. The studies we reviewed demonstrate that both motor and language recovery in chronic stroke are associated with oscillatory dynamics, primarily centered around beta and alpha bands. Although there are some differences in patterns of this dynamics between resting and active states, as well as between motor and language recovery, they can still be accommodated within the suggested framework, whose basic tenet is that the excitation–inhibition balance modulations may improve the efficacy of neural information processing, particularly related to top-down control abilities, common for motor and language tasks. These processes may, in turn, be tracked non-invasively using oscillatory brain dynamics. Due to the very limited number of studies available and discrepancies between them, this framework is still in need of substantial development and refinement that we will briefly discuss below.

### Motor and language recovery: Oscillatory effects and possible underlying mechanisms

In chronic motor stroke studies, resting-state oscillatory activity correlated with improvements differently, depending on the frequency band. In general, higher-frequency band activity, primarily beta, correlated positively with better recovery, while low-frequency bands (delta and theta) correlated positively with poorer recovery. Similarly, in chronic aphasia studies, resting-state data also showed negative correlations between oscillatory activity and functional improvements for lower (theta and delta) and positive for higher (alpha and beta) frequency bands.

Active-task studies, in turn, found recovery-associated higher-frequency power modulations during motor tasks related to the primary motor areas, both ipsi- and contralesionally. The modulations include both pre-movement desynchronization and post-movement oscillatory resynchronization, their amplitudes and durations. Further studies show recovery-associated modulations of task-dependent activity to also take place outside the primary motor cortex, involving, e.g., premotor, supplementary motor areas and wider frontoparietal networks. Similarly, in the active-state language studies, task-dependent higher-frequency beta and alpha modulations were observed mostly beyond the primary language perisylvian areas and their contralesional homologs. Furthermore, the available evidence suggests that a stronger decrease (ERD) of beta and alpha power during speech tasks might be associated with more successful recovery. These similar findings point to a distributed network nature of plastic recovery processes common for motor and language abilities, which may become manifest in modulations of rhythmic brain activity.

Considering the resting-state findings above, we might speculate that the recovery-related decrease of lower frequencies power and the corresponding increase of the higher-frequency activity (primarily beta) might reflect more successful functional reorganization, taking place before the onset of the stroke’s chronic stage. These higher frequency oscillations are associated with complex cognitive and behavioral processing (Pulvermüller et al., 1997), in contrast to the lower-frequency oscillations: an increase in perilesional lower-frequency oscillatory power, delta and theta, is known to correlate with more expressed dysfunction (Butz et al., 2004). In this view, this successful reorganization may be characterized by a more “optimal” resting excitatory–inhibitory balance achieved during the post-stroke cortical functional reorganization.

When considering active-state studies, their results generally suggest that task-dependent modulations of high-frequency oscillatory activity (primarily in beta and alpha bands) relate more to the greater functional efficiency of wide distributed neural networks during task-related performance. These effects, in contrast to the resting-state patterns, might be observed not only in perilesional, but also in intact extralesional areas. Furthermore, not just the presence of this extralesional activity, but rather its dynamic synchronization and desynchronization taking place online during task execution may imply more efficient excitation/inhibition balance, which enables such rapid network dynamics as the driver of recovery, optimizing cortical information processing (Yizhar et al., 2011).

The differences between resting and active state results might be, at least to some extent, related to the neurophysiological processes that underly different, yet not mutually exclusive, functional recovery mechanisms. These are (1) reduction of impairment (or functional restoration) and (2) compensation (Zeiler and Krakauer, 2013), both associated with inhibitory control of cortical excitation but mediated by two different GABAergic mechanisms: synaptic and extrasynaptic (Farrant and Nusser, 2005). Functional restoration occurs when the excitability of the perilesional tissues shifts toward the premorbid level. This might be achieved primarily by the decrease of tonic perilesional inhibition (Carmichael, 2012). This inhibition, driven by the extrasynaptic GABA activity, becomes increased due to the excessive post-stroke extracellular level of GABA in the perilesional tissues, as animal studies show (Clarkson et al., 2010). Such an inhibition initially plays a protective role, preventing the excitotoxicity leading to the neural cells’ death (Lipton, 1999). However, later on, by the chronic stage, it may become dysfunctional. The extrasynaptic tonic inhibition decreases the excitability of the pyramidal neurons and the interneurons’ synaptic inhibitory activity, or phasic inhibition (Carmichael, 2016). The phasic inhibition shapes the glutamate-driven firing of the excitatory neurons in the neural microcircuits generating oscillations, including beta and alpha (see the Introduction section). In turn, a release of phasic inhibition corresponds to restorative processes, i.e., the shift of excitation–inhibition balance toward the premorbid level and consequent functional improvements (Hiu et al., 2016). Together with the increase of the glutamate-driven excitation in the perilesional cortex, that may also enhance the functional recovery. Particularly, at the oscillatory level, this would be reflected in an increased beta and alpha activity and might be the reason why better recovery is associated with this activity synchronization in the resting state. In contrast, the compensatory mechanism might imply recruitment of intact cortical areas into carrying out the impaired functions. This involvement is manifest as altered task-dependent activity patterns observed in these areas. Such alterations would be caused by changes in the synaptic connections’ efficiency between excitatory and inhibitory cells (Vogels and Abbott, 2009; Zhou and Yu, 2018). Particularly, modulations of the synaptic GABAergic inhibitory activity (Kittler and Moss, 2003; Lüscher and Keller, 2004) might shift the functional excitatory–inhibitory balance toward the level corresponding to more efficient cognitive and behavioral performance (Sengupta et al., 2013; Doyon et al., 2016). In turn, this might be the cause of the observed modulations in the task-related oscillatory dynamics.

Hence, we suggest that the functional role of the oscillatory processes and their recovery-related modulations might differ depending on their origin either in proximal perilesional parts of the respective stroke-affected areas (language, motor) and their contralateral homologs, or in distal extralesional intact areas. That is, higher-frequency oscillatory modulations observed peri- and extralesionally might be driven by different mechanisms of the excitatory/inhibitory balance change. Perilesional functional recovery might be primarily driven by the release of the GABAergic extra-synaptic tonic inhibition and may be enhanced by an increase in glutamate-driven excitation. This is associated with the increase of the GABAergic synaptic phasic inhibition and synchronization of the higher-frequency oscillatory activity, primarily beta. In turn, compensatory motor and language recovery engaging intact extralesional areas might be primarily driven by the synaptic GABAergic signaling that provides short-living and, importantly, task-dependent phasic inhibition (Ward, 2017). The increase of this phasic activity may manifest in the task-dependent modulations of the beta and alpha activity that rely on the interactions between excitatory and inhibitory cells. Crucially, as the reviewed studies show, these oscillatory activity changes correlate with functional recovery.

### Compensatory recovery of motor and language abilities in chronic stroke: The role of top-down control mechanisms

However, the exact functional mechanism underpinning compensatory recovery remains obscure. The results of the motor and language studies reviewed might indicate that this compensation could be driven by the involvement of the domain-general neural networks supporting working memory and cognitive control processes. Within the framework of the top-down control mechanism suggested in the Introduction, beta and alpha oscillations are generated by the reciprocally connected excitatory and inhibitory neurons that form microcircuits lying in the deep cortical layers (primarily, L5) and sending projections both to more superficial layers and to other cortical areas. Recovery-related modulations of beta and alpha activity may stem from rebalancing the synaptic strengths within the recruited neural microcircuits (Spitzer and Haegens, 2017) which affects the networks’ task-induced excitatory–inhibitory dynamics. Hence, these modulations might reflect the increased information processing capacity within distributed neural networks, possibly as a result of synaptic plasticity processes driving the functional recovery.

The processes of interactions between deep and superficial cortical layers might be the basis of the cortical mechanism of task-dependent synchronization and desynchronization (Bastos et al., 2018; Miller et al., 2018). In this view, a greater inhibitory impact from deep cortical layers causes beta and alpha synchronization in superficial layers. In contrast, the decrease of this inhibitory impact leads to beta and alpha desynchronization. Physiologically, beta power suppression (beta-ERD) in these networks might reflect a decrease in cortical inhibition and a corresponding increase in cortical excitation (Pfurtscheller, 2006). This pattern, whilst possibly universal, seems most specific to associative cortical areas^1^. Moreover, within this model, greater beta and alpha desynchronization (leading to cortical excitation increase) might reflect a greater working memory load during top-down controlled retrieval of target representations (Miller et al., 2018). The opposite process, beta, and alpha synchronization might indicate a decrease in working memory load. Importantly, these beta and alpha power modulations are accompanied by the opposite modulations of gamma power observed in the superficial cortical layers and reflecting the bottom-up sensory inputs’ activity (Wang, 2010). In other words, the task-dependent beta and alpha power modulations might reflect the mechanism of the working memory control, particularly the dynamics of its capacity.

Notably, recovery-related oscillatory effects in beta/alpha bands observed in the reviewed active-task studies, occur not only in perilesional language primary and motor sites or their contralateral homologs, but also in other areas, that include mostly frontal and parietal regions. This frontoparietal topography is typical of a top-down executive control network (Woolgar et al., 2011; Fedorenko et al., 2013; Shashidhara et al., 2019). The alteration of the task-related excitation–inhibition dynamics in these areas during motor and language tasks performance might support the increased load on the domain-general cognitive processes, such as working memory, selective attention and executive control. This increased load might be considered as a compensatory mechanism that provides additional cognitive resources for stroke patients to perform complicated motor or language tasks. Hence, beta and alpha modulations within the intact associative cortical areas might be a *biomarker* that reflects compensatory mechanisms both at neurophysiological and cognitive levels.

There is indeed evidence that working memory dysfunction may be a core deficit in aphasia (Amici et al., 2007; Mayer and Murray, 2012; Wright and Fergadiotis, 2012). Moreover, working memory and cognitive control processes are tightly involved in both language and motor performance in healthy individuals (Engel and Fries, 2010; Piai and Zheng, 2019). However, less is known about this link for motor stroke patients, though motor and language recovery processes after stroke are known to be clinically correlated, as discussed above and in previous studies (Gialanella and Ferlucci, 2010; Ginex et al., 2017). In this respect, the studies by Buch and colleagues and by Ray and colleagues shed more light on this link as their results suggest that the recovery-related oscillatory effects they found might be associated with the motor control ability as a recovery factor (Buch et al., 2012; Ray et al., 2020). Hence, the recovery-related oscillatory modulations might reflect those aspects of working memory that support top-down cognitive control, as there is evidence of a common evolutionary origin for both speech and motor skills (Chatham and Badre, 2015).

In sum, task-dependent modulations of beta and alpha-band activity generated in intact extralesional cortical areas might reflect a common compensatory mechanism of both motor and language functional recovery in chronic stroke. The alteration of excitation and inhibition in these areas during tasks performance might support the increased load on the domain-general top-down control processes. The results of the studies on the task-dependent oscillatory effects related to the chronic stroke recovery provide arguments in favor of our suggestion that beta/alpha modulations reflecting functional excitatory–inhibitory balance dynamics, associated with the top-down control processes, might be a biomarker of both motor and language recovery in the chronic stroke (see Chart 2 in Supplementary material).

### Variability of top-down control mechanisms and underlying neural networks

Notably, the recovery-related oscillatory modulation patterns might be rather different, which may stem from diversity in lesions as well as from the highly complex nature of neurocognitive processes and functions involved, including, importantly, working memory. It is known to provide many different tools and strategies for manipulating multidomain information, including encoding, maintenance, retrieval, execution, updating, etc. within different tasks under variable cognitive demands (Fuster, 2009; Nyberg and Eriksson, 2016). These mechanisms include working memory-intense operations, such as selective attention, guided search, retrieval, action planning, control, etc. that are crucial for complex goal-directed behavior, e.g., voluntary action and speech. Many of them are known to be associated with oscillatory processes in beta band, related to the cortical top-down projections and inter-areal coherence they support (Wang, 2010). These oscillatory processes supporting top-down control of the working memory processes coordinate the neural activity between basal ganglia and cortical (mostly prefrontal) areas, modulated by the nigrostriatal dopaminergic system (O’Reilly and Frank, 2006). Functionally, this cortico-basal system subserves control components of voluntary movement execution (Pfurtscheller et al., 2003). An impairment of this control occurs in Parkinson’s disease, a disorder that involves both motor and cognitive symptoms (Bastiaanse and Leenders, 2009) and is associated with beta-band activity reduction. Cortico-basal loops are associated with voluntary, goal-directed control of motor output in various tasks, which is reflected in beta-ERS/ERD dynamics.

In other words, the oscillatory activity that supports top-down control processes might be driven by different neural networks. These might be frontoparietal, cortico-basal, cingulo-opercular and probably other networks and nodes (Dosenbach et al., 2008). Moreover, beta and alpha oscillatory activity in different attention and memory tasks is known to be modulated by norepinephrine- and cholinergic systems (Benchenane et al., 2011; Iemi et al., 2022). Importantly, these neurotransmitter systems are known to be involved in reinforcement and learning processes (Pennartz, 1995), and their activity might be an additional factor that impacts experience-induced plasticity in stroke recovery.

Consequently, the top-down control mechanisms and the underlying neural activity might be influenced by a multitude of factors which, in turn, determines the variability observed in recovery patterns. The specific mechanisms underpinning this influence remain unknown and require further research. Particularly, the functional meaning of the excitation–inhibition balance and its dynamics within various top-down control networks, the neural correlates of this dynamics (particularly oscillatory events) need more systematic investigation both in healthy individuals and in stroke patients.

### Training-dependent beta and alpha oscillatory dynamics and individual recovery strategies

As we hypothesized in the Introduction, *training-induced* modulations of beta/alpha oscillatory activity within distributed functional neural networks associated with top-down control abilities might also predict motor and language rehabilitation outcomes. However, the compensatory oscillatory dynamics, known from studies of stable patients reviewed above, have not yet been systematically investigated in chronic stroke patients undergoing behavioral training. Some hints can still be drawn from data obtained in healthy controls. It has been shown that oscillatory activity, especially in beta band, is sensitive to training as it reflects experience-dependent plasticity both in sensorimotor and in higher-order cognitive systems (Bavelier et al., 2010), with different patterns of oscillatory dynamics correlating with performance at different stages of skill acquisition. It can also be modulated by reinforcement and reward (Trilla Gros et al., 2015), an essential part of forming novel associations in the learning process.

In motor learning, an increase of beta desynchronization before voluntary movement at the early learning stages might reflect the need for adaptive modifications of cortical motor representations that occur as a result of backward afferent updates in healthy individuals (Herrojo Ruiz et al., 2017). In similar vein, beta modulations have been shown to take place in acquisition and consolidation of lexical-semantic representations for novel words (Bakker et al., 2015). Furthermore, both an increase and a decrease in beta power after performing a motor task can correlate with successful learning, depending on the individual learning strategy (Haar and Faisal, 2020). This indicates that excitatory–inhibitory processes reflected in oscillatory modulations might play different functional roles at various stages of complex skills training, and possibly during the recovery. The latter has been shown in clinical subgroups of stroke patients, who demonstrated different recovery patterns dependent on the initial alpha-ERD levels (Ray et al., 2020). The variability of recovery-related ERD patterns may derive from the individual differences in the task-induced cognitive load across patients having different impairments’ severity. On the one hand, this might point to the compensatory increase of the task-related information processing efficiency. On the other hand, it likely points to the increased cognitive efforts that stroke patients have to make when performing complex motor and language tasks.

This approach may potentially resolve some controversies between the results of the studies included in the current review. Particularly, the motor studies report synchrony/connectivity modulations associated with recovery, both in beta band and in other frequencies. However, the direction of these modulations varies across studies. Some of them show that an increase of oscillatory activity parameters (i.e., ERD, ERS, or their duration) is associated with better recovery, while others show opposite trends. Since beta/alpha oscillatory power modulations are sensitive to training, the observed differences are likely related to plastic reorganization processes linked to re-tuning excitatory–inhibitory balance across distributed cortical areas. Correlations between oscillatory power modulations (beta or alpha) with behavioral improvements may vary across patients because they rely on different strategies of motor training and action execution. Clearly, experience-induced oscillatory modulations might be affected by a variety of factors, and substantial further investigations are needed to validate their potential as biomarkers of recovery.

## Limitations and further directions

There are various limitations of the suggested framework and of the present interpretations of available findings, which will need clarification in future research. First, the present review is mostly focused on the compensatory recovery mechanism driven by the recruitment of the intact extralesional areas and reflected in modulations of their oscillatory activity in beta and alpha bands. However, interactions with other recovery mechanisms were left mostly beyond the scope of this review and remains to be tackled. For instance, substantial research is needed to clarify the relative contributions of the recovery-related dynamics in the primary functional areas vs. associative cortical ones. Moreover, we have emphasized that the recovery driven by the top-down control system might rely on multiple functional and neural mechanisms, different aspect of which need systematic and comprehensive investigation.

Also, based on the above findings, it stands to reason that efficient functional recovery of motor and language abilities in chronic stroke is achieved when an optimal task-dependent excitatory–inhibitory balance in the specific neural networks is established as an outcome of interventions (i.e., behavioral training). Whereas the available data do not allow for unequivocal confirmation of this suggestion, future studies could pursue this in several ways. For instance, they will need to clarify which particular spatial and temporal patterns of oscillatory modulations may predict more successful recovery. They will also need to take into consideration the input of a variety of individual clinical and demographic variables.

More specifically, studies are needed that could more systematically investigate *training-induced* changes in task-dependent oscillatory dynamics in chronic stroke. At present, evidence is particularly lacking for PSA, where more complex metrics of oscillatory activity (e.g., not only power, but also duration, frequency, and phase dynamics) are crucial for understanding its functional relevance for recovery. To scrutinize these dynamics, neuroimaging techniques with high spatial and temporal precision, such as MEG with individual MRI-based source analysis (taking into account conductor properties of lesioned tissues, which is not a trivial challenge) seem optimal.

This future work should consider that the patterns of cortical oscillatory modulations might also vary depending on rehabilitation training techniques and on tasks used to measure behavioral outcomes. Finally, it seems essential to define individual patients’ characteristics affecting the specific dynamics of these processes and their exact associations with better recovery. Ultimately, this knowledge should contribute to better strategies for diagnoses, therapies, and optimal rehabilitation techniques for chronic stroke patients.

## Conclusion

To sum up, we reviewed the evidence that neural excitation/inhibition balance reflected in oscillatory activity modulations may be a candidate biomarker of functional recovery in chronic stroke. Among different frequency bands, modulations of beta/alpha activity might be most relevant for motor and speech recovery in chronic stroke patients. Based on the limited evidence available, we suggest that this activity might be associated, among others, with domain-general processes of top-down control, such as working memory, selective attention, and executive control, which are involved in both training and performance in motor and language tasks. This interpretation of shared recovery mechanisms and respective biomarkers goes well in line with the established notion of the evolutionary close relationship between neural underpinnings of motor and language functions. Based on this, the following tentative conclusions can be made:

• Motor and language beta and alpha activity modulations are associated with chronic post-stroke recovery.
• These modulations might reflect the recovery-related dynamics of the excitation–inhibition balance, both in perilesional and, importantly, in extralesional intact cortical areas.
• Functionally, the task-dependent modulations in beta and alpha bands may be related to a compensatory involvement of the domain-general top-down control mechanisms, common for motor and language recovery.
• Beta and alpha activity modulations in language and motor tasks might thus be a common biomarker of language and motor improvements in chronic stroke.
• Beta and alpha oscillatory dynamics might reflect the efficiency of motor and language training in chronic stroke rehabilitation.

The latter suggestion requires substantial future research which should both explore the mechanisms underlying these dynamics and validate its potential practical utility as a training-induced recovery biomarker.

## Author contributions

MU reviewed the literature and prepared an earlier version of the manuscript. MU and YS discussed the review and edited the manuscript. Both authors contributed to the article and approved the submitted version.

## Footnotes

1 Notably, this working-memory model suggests functional roles not only for beta activity but also for alpha, gamma, and theta bands (Miller et al., 2018) which have indeed been implicated by several studies reviewed above.
