# Supplementary material for: Oscillatory beta/alpha band modulations: A potential biomarker of functional language and motor recovery in chronic stroke?
Source: Front Hum Neurosci. 2022 Sep 26;16:940845. doi: 10.3389/fnhum.2022.940845 (PMC9549964; doi:10.3389/fnhum.2022.940845)
Supplement: Supplementary file 1 [file Data_Sheet_1.pdf]

Chart 1. A framework: multilevel mechanisms of top-down control in motor and language functions

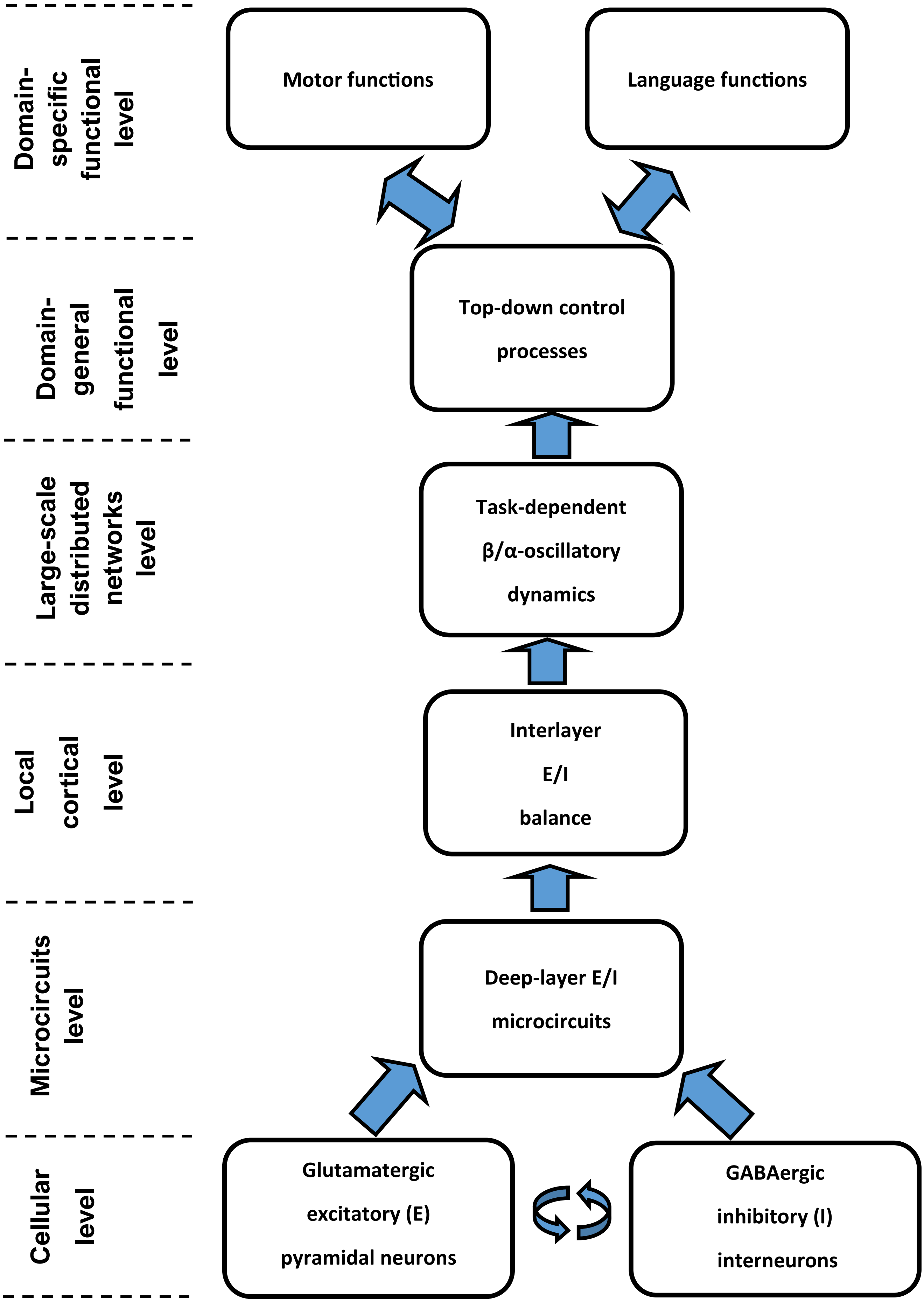

Chart 1 legend

Cellular level: Deep-layer excitatory glutamatergic and inhibitory GABAergic cells and their synaptic connections

Microcircuits level: Neural excitatory-inhibitory circuits - possible generators of beta and alpha oscillatory activity (Jensen, 2005; Jensen & Mazaheri, 2010)

Local cortical level: Axonal projections from the deep (L5) to the superficial (L2/3) cortical layers locally modulate cortical excitation/inhibition balance via beta/alpha synchronization/desynchronization (Bastos, 2018)

Distributed neural networks level: Beta/alpha oscillatory modulations in the distributed neural networks are associated with complex motor and language tasks performance (Schmidt, 2018; Piai & Zheng, 2019)

Domain-general functional level: Task-dependent beta/alpha modulations across wide cortical areas might support domain-general top-down control processes (i.e., working memory control, see: Miller, 2018)

Domain-specific functional level: Domain-specific motor and language functions might share a domain-general mechanism of top-down control for complex behavioral tasks performance (see the Introduction part of the current review)

Chart 2. Putative relationships between functional levels and potential recovery indices

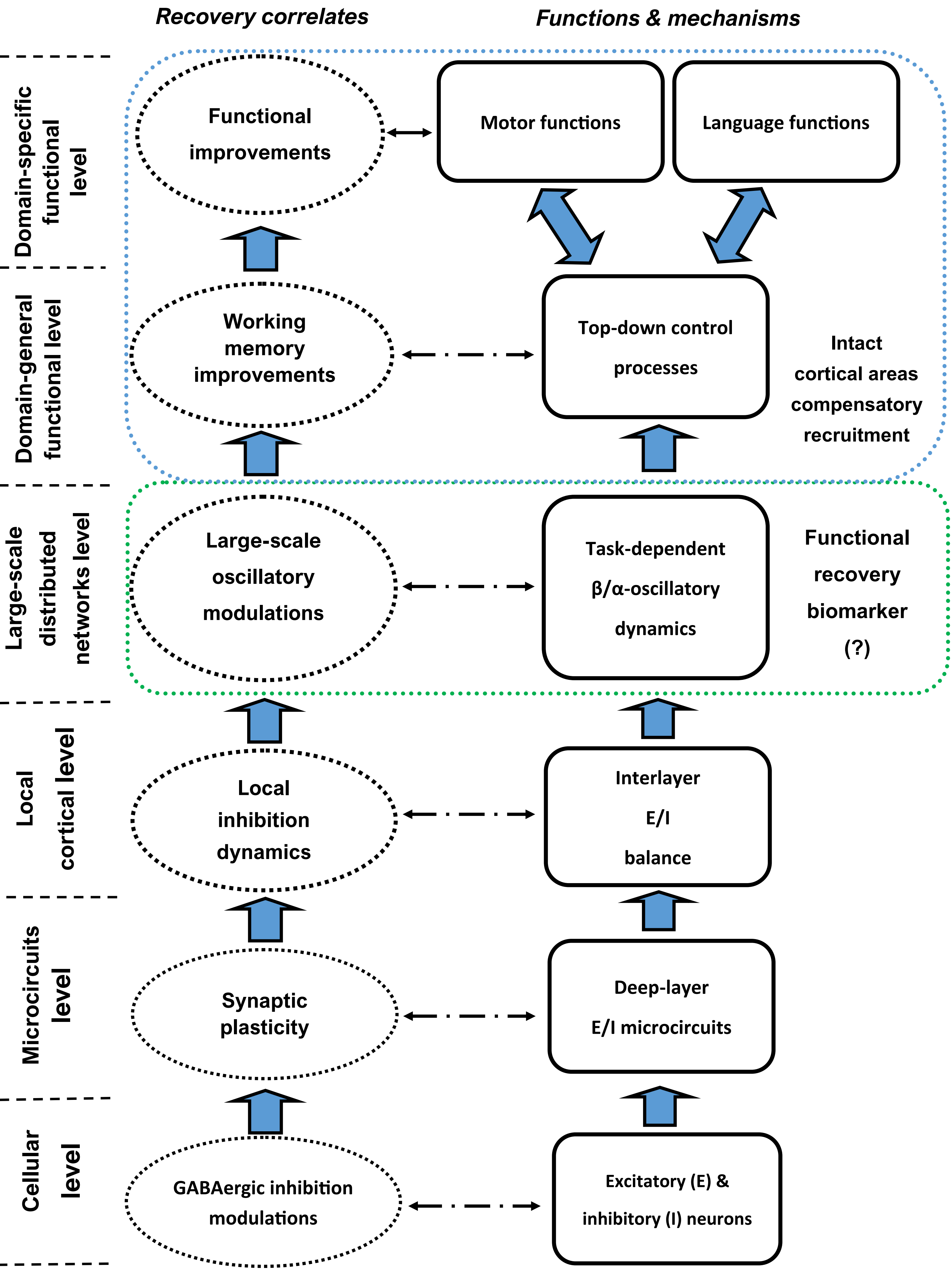

Chart 2 legend

***Cellular level**:* GABAergic interneurons' inhibitory activity modulations are associated with better functional recovery (Ward, 2017)

***Microcircuits level**:* the synaptic plasticity within excitatory/inhibitory circuits might improve the information processing efficiency (Zhou and Yu, 2018)

Local cortical level: the local cortical excitatory-inhibitory dynamics impact the functional recovery in stroke (Carmichael 2012)

***Distributed neural networks level**:* beta and alpha oscillatory dynamics across the cortex (driven by alteration of excitatory-inhibitory balance) are associated with motor and language recovery in chronic stroke patients (see the results of studies included in the current review and summarized in the Supplementary Tables 1 and 2). Hence, these oscillatory dynamics might be a candidate biomarker of functional recovery in stroke.

***Domain-general functional level**:* task-dependent beta/alpha oscillatory power dynamics (mostly ERD/ERS in the recruited intact cortical areas) might reflect the greater involvement of the domain-general top-down control processes (i.e., working memory control, see Miller, 2018) into domain-specific motor and language tasks in stroke patients

***Domain-specific functional level**:* the shared mechanism of top-down control might play a compensatory role, common for motor and language post-stroke recovery (see the Discussion part of the current review)

Supplementary Table 1. Summary of studies on chronic motor stroke recovery and related oscillatory effects

N	Study	Subjects	Training procedures	Evaluation measures	Experimental paradigm	Outcomes highlights
**Resting-state motor studies**						
1	Hordacre et al., 2020	N=36 chronic stroke patients, N=25 healthy controls	None	Action Research Arm Test (ARAT), Fugl-Meyer motor assessment (FMA), TMS measurements of MEP (motor-evoked potentials)	Resting-state EEG	Patients with better TMS-induced MEP responses had stronger beta resting-state functional connectivity (RSFC) for sensors positioned over sensorimotor regions. Beta-band (14-30 Hz) RSFC for these patients correlated positively with better upper limbs behavioral score
2	Thibaut et al., 2017	N=55 chronic motor stroke patients	None	Fugl-Meyer motor assessment (FMA), TMS measurements of motor thresholds (MT)	Resting-state EEG	High-frequency bands power spectrum in high alpha (10-13 Hz), low beta (13-20 Hz), and high beta (20-30 Hz) bilaterally correlated with better FMA scores.<br>High-beta (21-30 Hz) power increase in electrodes over central regions predicted better motor functioning (FMA): negatively in the affected hemisphere, positively in the unaffected hemisphere.<br>MT on the affected body side negatively correlated with high beta (21-30 Hz) power in the affected hemisphere
3	Saes et al., 2019	N=21 chronic motor stroke patients, N= 11 matched controls	None	Fugl-Meyer motor assessment (FMA), National Institutes of Health Stroke Scale (NIHSS), Motricity Index of upper and lower extremities (MI-UE, MI-LE)	Resting-state EEG	Brain symmetry index (BSI) for delta (1-4 Hz) and theta (4-8 Hz) bands was stronger in patients than in controls and negatively correlated with upper extremities motor performance scores (FMA). This spectral band power was increased in the affected comparing to the unaffected hemisphere.

**Motor task studies**						
4	Shiner et al., 2015	N=10 chronic motor stroke patients	None	Wolf-Motor Function Test timed-tasks (WMFT-tt), grip strength task, Fugl-Meyer Assessment, timed finger-tapping speed task, Motor Activity Log Quality of Movement scale (MALQOM), Box and Block Test (BBT), TMS measurements of MEPs (motor-evoked potentials)	Unilateral finger-tapping task, MEG	Stronger beta-ERD/ERS (13-30 Hz) amplitudes in motor and premotor cortices with a greater ipsilesional hemisphere involvement correlated with better motor functioning.<br>Beta-ERD lateralization index negatively correlated with motor functions scores.<br>Beta-ERD duration on the more affected site correlated negatively with motor functions scores.<br>Beta-ERS duration correlated positively with better motor functions and motor thresholds.<br>Ipsilesional beta-ERS peaks correlated positively with motor functions score.
5	Espenhahn et al., 2020	N=16 chronic motor stroke patients, N=20 matched controls	Training of wrist flexion and extension movements (affected arm in patients, non-dominant in controls)	Handedness Edinburgh test, grip strength test, Nine-Hole Peg Test (NHPT), SART, Motor performance accuracy in continuous tracking task	EEG, visual-cued wrist movement task: once before and twice after training	Post-movement beta rebound (PMBR), event-related synchronization in 10-25 Hz (beta/alpha) over the affected sensorimotor cortex immediately after training was associated with better motor performance 24h after training.<br>Learning rate was diminished in patients compared to healthy controls.
6	Wilson et al., 2011	N=4 chronic motor stroke patients	Intensive goal-directed motor 2-weeks training of the impaired hand + paired peripheral nerve stimulation (PNS, active/sham)	Wolf Motor Function Test (WMFT), the Action Arm Research Test (ARAT), Fugl-Meyer Assessment (FMA)	MEG, unilateral finger tapping task during (before and 3 weeks after training)	Post-movement beta-ERS (16-28 Hz) reduced in bilateral precentral gyri and SMA after treatment.<br>Pre-movement gamma-ERS (74-86 Hz) decreased in the affected precentral gyrus after treatment.<br>Post-movement beta-ERS in the affected hemisphere after treatment negatively correlated with FMA scores. Same non-affected precentral gyrus activity negatively correlated with the Action ARAT scores. Gamma-ERS over affected precentral gyrus after treatment negatively correlated with WMFT.

7	Buch et al., 2012	N=8 chronic stroke patients	2-3 weeks daily training sessions with sensorimotor brain–computer interface and hand orthosis device	Modified Ashworth Scale (MAS), Mini-Mental State Examination (MMS), grasp motor skill evaluation	MEG, brain-computer interface biofeedback task on sensorimotor mu-rhythm modulations	Graph measure of global functional network cost efficiency was positively associated with measures of sensorimotor mu rhythm in alpha (9-12 Hz) and beta (20-24 Hz) bands.<br>Structural integrity measures in ipsilesional frontoparietal were positively associated with sensorimotor rhythm modulation skill. The contralesional structural integrity measures of superior longitudinal fasciculus positively correlated with this skill.
8	Ray et al., 2020	N=22 chronic motor stroke patient	Robotic orthosis device training guided by self-modulations of sensorimotor mu-rhythm via proprioceptive brain-machine interface	Combined modified Fugl-Meyer assessment (cFMA)	EEG recordings across sessions of paretic hand movements training	Motor improvements (cFMA scores) in patients were associated with alpha-ERD (8-12 Hz) interhemispheric dynamics over bilateral central and parietal electrodes. Different initial interhemispheric alpha-ERD asymmetry predicted different motor-task strategies. Patients with initially strong postlesional alpha-ERD improved when ERD amplitude increased. Patients with initially small postlesional alpha-ERD improved when ERD amplitude decreased.<br>Generally, better clinical improvement correlated with alpha-ERD shift towards ipsilesional hemisphere.

Supplementary Table 2. Summary of studies on chronic post-stroke aphasia recovery and related oscillatory effects

N	Study	Subjects	Training procedures	Evaluation measures	Experimental paradigm	Outcomes highlights
**Resting-state speech studies**						
8	Nicolo et al., 2015	Two stroke groups (subacute-to-chronic): N=24 and N=18; N=26 age-matched controls	Individual multidisciplinary rehabilitation program combined with pharmacological treatment	Fugl-Meyer Assessment; Nine Hole Peg Test; stroke rehabilitation assessment of movement (STREAM) instrument; Geneva Bedside Aphasia Score (GeBAS)	Task-free EEG recording at the end of sub-acute and at the beginning of the chronic period	Beginning of sub-acute stage: motor and language clinical improvements correlated positively with global functional connectivity in beta band in the left and in theta band in the right hemisphere. Beginning of chronic stage: same measures correlated negatively with motor and language improvements. Correlations with motor and language improvements were specific to connectivity of primary motor cortex and Broca's area, respectively.
1	Dalton et al., 2021	N=19 chronic stroke patients, N=24 healthy controls	None	Western Aphasia Battery - Revised (WAB-R), Language Experience & Proficiency Questionnaire (LEAP-Q), Repeatable Battery for Assessment of Neuropsychological Status (RBANS), Wechsler Intelligence Scales - Picture Completion, Apraxia Battery for Adults - 2 (ABA-2). Aprosodia Battery; Discourse Production Test, Boston Naming Test, Discourse Comprehension Test,	Resting-state EEG	Eyes-open condition: greater beta power in patients than in controls. MC language scores positively correlated with beta (~13-30 Hz) power (whole-brain/left-lateralized). RBANS scores positively correlated with left-hemispheric beta power.<br>Eyes-closed condition: lower beta and higher theta (~ 4-7 Hz) power in patients than in controls. MC scores in patients positively correlated with left-hemispheric beta and alpha (~8-12 Hz) power and negatively with whole-brain theta power (different montages). Resting state oscillatory patterns showed various test-retest reliability across frequency bands.

				Main Concept (MC) analysis		
2	Rozelle and Budzynski, 1995	Single-case study: chronic post-stroke aphasia patient	Neurofeedback training of activity modulation in speech and sensorimotor areas: theta (4-7 Hz) decrease and low beta (15-21 Hz) increase	Boston Aphasia Severity Rating Scale, Boston Naming Test, Apraxia Battery, Stroop Color and Word Test, Brief Symptom Inventory, Conners Continuous Performance Test	Resting state EEG before and after training	Post-treatment decreases of theta (4-7 Hz) activity over left frontal, central, parietal regions, and increase of beta (15-18 Hz) activity over midline and frontal regions. Improvements in neuropsychological and speech measures (speech fluency and word finding).
3	Meinzer et al., 2004	N=28 chronic aphasia patients, N=25 healthy controls	Intensive 10-day training using Constraint-Induced Aphasia Therapy (CIAT)	Aachen Aphasia Test (AAT), Token Test	MEG task-free recordings before and after therapy	AAT language scores increased after speech therapy for entire group. Delta activity was left-lateralized before and after therapy. Improvements were associated with left-hemispheric delta (1-4 Hz) power decrease in 16 patients and delta power increase in 12 patients. Left-hemispheric (but not right-hemispheric) delta power change correlated positively with improvements in AAT scores
**Speech task studies**						
4	Spironelli et al., 2013	N=13 chronic aphasia patients, N=11 healthy controls	None	Aachen Aphasia Test (AAT)	EEG, paired visual word stimuli, word-matching tasks: semantic, phonological, orthographic	At the anterior electrodes the beta-activity (21-28 Hz) was left-lateralized for orthographic and phonological tasks. At the central electrodes it was right-lateralized for semantic and phonological tasks and bilateral for orthographic task. Only left posterior high beta (21-28 Hz) percentage correlated with behavioral and clinical measures. Negative correlations between

						semantic and phonological scores and high beta band percentage.
5	Meltzer et al., 2013	N=25 chronic post-stroke aphasia patients, N=24 healthy controls	None	Western Aphasia Battery (WAB), Psycholinguistic Assessments of Language Processing in Aphasia (PALPA), Psycholinguistic Assessment of Language (PAL), sentence-picture matching task	Sentence picture-matching task (PAL) implemented in MEG paradigm	Task-related ERD in 8-30 Hz band was more right-lateralized for patients. Increased activity in the right hemisphere was in parietal, temporal, and frontal regions. Bilateral ERD pattern in posterior temporal and parietal areas for sentence comprehension. Right-lateralized pattern during task delay period in the right superior, middle frontal gyri, in the superior and inferior parietal lobes. ERD strength in bilateral posterior temporal and parietal regions correlated with better comprehension during sentence presentation. ERD in right superior and middle frontal gyri, superior and inferior parietal lobes correlated with performance during memory delay.
6	Kielar et al., 2016	N=19 chronic post-stroke aphasia; N=19 healthy age-matched controls	None	Montreal Cognitive Assessment, fluency and discourse tests, Psycholinguistic Assessments of Language Processing in Aphasia (PALPA), Boston Naming Test, Western Aphasia Battery (WAB)	MEG, sentence comprehension paradigm with varying semantic and syntactic sentence features	Differences between patients and controls were found in task-related beta/alpha-ERD (8-30 Hz). For semantic tasks left-hemispheric activation was smaller for patients in ventral frontotemporal, anterior temporal and temporooccipital areas. Right-hemispheric temporal beta/alpha-ERD in right superior, middle and inferior temporal gyrus correlated with semantic tasks accuracy. For syntactic tasks, patients showed lower activation than controls in frontal, posterior temporal and dorsal parietal areas. Bilateral temporal beta/alpha-ERD in right temporoparietal cortex, superior temporal gyrus, precuneus, middle frontal gyrus and left inferior

						parietal lobule correlated with semantic accuracy.
7	Piai et al., 2017	N=6 chronic aphasics; N=6 healthy controls	None	Western Aphasia Battery (WAB)	EEG, spoken sentence completion task with two conditions: constraint and unconstraint completion	Significant context-specific effects were found in time interval between sentence presentation and target picture stimulus in beta/alpha band (8-25 Hz). Beta/alpha-ERD was left-lateralized in controls, right-lateralized in patients and absent for poor responders among patients.
